# Coexpression Network Analysis of lncRNA Associated with Overexpression of DNMT1 in Esophageal Epithelial Cells

**DOI:** 10.1155/2021/7162270

**Published:** 2021-10-08

**Authors:** Yi Lei, Yi Xu, Xu-feng Li, Yan Chen

**Affiliations:** ^1^The Medical School of Jiaxing University, Jiaxing, China; ^2^Department of Health Toxicology, College of Public Health, Xinjiang Medical University, Urumqi, China; ^3^Jiaxing Center for Disease Control and Prevention, Jiaxing, China

## Abstract

Screening and preliminary identification of high DNMT1 expression-related lncRNA, which is involved in various interrelated signaling pathways, has led to the development of a theoretical basis for various types of disease mechanisms. Differential expression profiles of lncRNA and mRNA were identified in a microarray. Ten lncRNAs with high levels of variation were identified by qRT-PCR. KEGG and GO analyses were used to identify differentially expressed mRNAs. Six signaling pathways were selected based on the KEGG results of the lncRNA-mRNA expression network analysis. From the microarrays in the experimental and control groups, we found a total of 6987 differentially expressed lncRNAs, and 7421 differentially expressed mRNAs were obtained (*P* < 0.05; fold change > 2.0x). GO analysis and KEGG pathway analysis showed high expression of DNMT1 in esophageal epithelial cells. Nine pathways were involved in mRNA upregulation, including natural killer cell-mediated cytotoxicity and many other prominent biochemical pathways. Forty-six pathways were associated with downregulated mRNAs and ribosomes involving multiple biological pathways. Coexpression network analysis showed that 8 mRNAs and 16 lncRNAs were linked to the p53 signaling pathway. In Helicobacter pylori infections, interactions occurred between 22 lncRNAs and 11 mRNAs in the ErbB signaling pathway and between 19 lncRNAs and 8 mRNAs in epithelial cell signal transduction. Interactions were present between 19 lncRNAs and 5 mRNAs in the sphingolipid signaling pathway, along with interactions between 21 lncRNAs and 12 mRNAs in the PI3K-Akt signaling pathway. Cytotoxicity interactions occurred between 22 lncRNAs and 9 mRNAs in natural killer cells.

## 1. Introduction

Epigenetics is the study of genetic changes in gene activity or function and does not involve changes in the DNA sequence itself. Its molecular mechanisms include DNA methylation, chromosome modification, histone modification, and RNA interference. Historically, DNA methylation was discovered in mammals long before the DNA was identified as genetic material [1, 2]. DNA methylation is accomplished by transference of methyl groups from S-adenosylmethionine to the 5′ position of cytosine via DNA methyltransferase activity (DNMTs). Three catalytically active DNMTs have been identified in mammals: DNMT1, DNMT3a, and DNMT3b [3]. DNMT1 is the most important enzyme for maintaining DNA methylation status in vertebrates and is also one of the most well-known enzymes. It can play a role in gene silencing as well as DNA methylation repair [4]. High DNMT1 expression levels can cause methylation pattern variations that result in silencing of tumor suppressor genes and oncogene activation. Abnormal DNMT1 activity can lead to prostate [5–7], lung [8, 9], kidney [10–12], and bladder cancer [13–16]. Obviously, the integrity of the DNA methylation system is critical to the health of mammals.

Long noncoding RNA (lncRNA) is a form of RNA lacking open reading frames and does not encode proteins. The transcripts are more than 200 bp in length and are found in the nucleus or cytoplasm. A large number of studies have reported that lncRNA plays an important role in the development of many diseases. It has tissue, cell, developmental, spatiotemporal, and disease-related specificity and is widely involved in cell differentiation and metabolism. Cell proliferation occurs in the course of various diseases where normal lncRNA function is altered [17–24]. Differential expression of lncRNA exhibits tumor specificity, is not affected by other factors, and can be used as an independent tumor-specific predictor [25]. Studies have shown that lncRNA is an important regulatory factor in the human genome that can control DNA methylation and histones as an epigenetic modulator and transcriptional and posttranscriptional regulator in a cis or trans manner. These activities include modification and chromatin remodeling to silence or activate genes [26–28].

Since lncRNA does not encode proteins, it appears to act indirectly as compared to the direct action of mRNA. Therefore, coexpression analysis is widely used to elucidate the relationship between lncRNAs and messenger RNA (mRNAs) actions [29, 30]. It can reveal key lncRNAs and help to elucidate new regulatory mechanisms.

In the present study, using a previously developed high-expression DNMT1 cell line and a normal esophageal epithelial cell line, we used the Agilent Human lncRNA V5 chip technology to screen differentially expressed lncRNA and coexpressing them. The analysis included an in-depth evaluation of specific lncRNA functions that can form a foundation for an in-depth examination of disease mechanisms.

## 2. Methods

### 2.1. Samples

The experimental groups chosen for this study included a high expression DNMT1 cell line [31], which was developed following transfection of a WV0132 plasmid using TALE technology. The control group was a normal esophageal epithelial cell line HEEC.

### 2.2. lncRNA Microarrays

The Agilent Human V5 Microarray analysis (Agilent, USA) was performed using a Gene Expression Hybridization Kit (Agilent USA) according to the manufacturer's instructions. Slides were washed in staining dishes with a Gene Expression Wash Pack (Agilent, USA) and scanned by an Agilent Scanner G2505C (Agilent, USA) with default settings according to the manufacturer's instructions. Raw data were normalized by the quantile algorithm using Gene Spring Software 13.1 (Agilent Technologies).

### 2.3. Quantitative Real-Time Polymerase Chain Reaction (qRT-PCR)

We selected 10 differentially expressed genes to evaluate their activity in overexpressing DNMT1 and esophageal epithelial cells. Total RNA was isolated from all samples using a mirVanaTMRNA Kit (Ambion, USA) then reverse transcribed using a Quick Amp Labeling Kit, One-Color (Agilent, USA) according to the manufacturer's instructions. qRT-PCR was performed using a QuantiFast® SYBR® Green PCR Kit (Qiagen, Germany). Glyceraldehyde 3-phosphate dehydrogenase (GAPDH) was used as an internal control. Primer sequences are shown (Table 1).

### 2.4. Statistical Analysis

Data were analyzed using SPSS (version 17.0; SPSS Inc., Chicago, IL, USA). Differentially expressed genes or lncRNAs were then identified by fold change as well as *P* value levels calculated by *t*-test. The threshold set for up- or downregulated genes was ≥2.0 times the median value and a *P* value of ≤0.05. lncRNA-mRNA coexpression networks were constructed using Cytoscape software 54 (version 3.4.0; The Cytoscape Consortium, San Diego, CA, USA).

## 3. Results

### 3.1. Identification of Differentially Expressed lncRNAs and mRNAs

**Figure 1:**
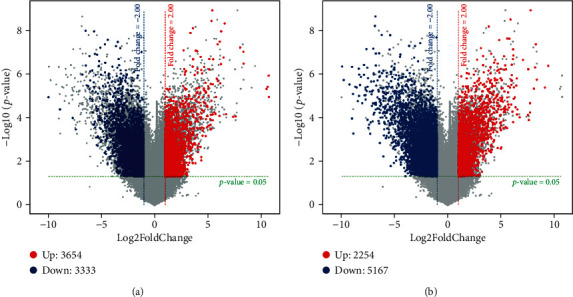
Volcano plot of differentially expressed lncRNA and mRNA in high-expression DNMT1 cell lines and normal esophageal epithelial cell lines. (a) Volcano plot-lncRNA; (b) volcano plot-mRNA. Red dots represent upregulated lncRNAs; blue dots represent downregulated lncRNAs (*P* < 0.05; fold change > 2.0).

There were 6987 lncRNAs that were differentially expressed from the microarrays of experimental and control groups. Of those, 3654 were upregulated and 3333 were downregulated. In addition, 7421 differentially expressed mRNAs were identified that included 2254 that were upregulated and 5167 that were downregulated. A volcano plot was created to identify differences among the various lncRNAs and mRNAs (Figure 1).

**Figure 2:**
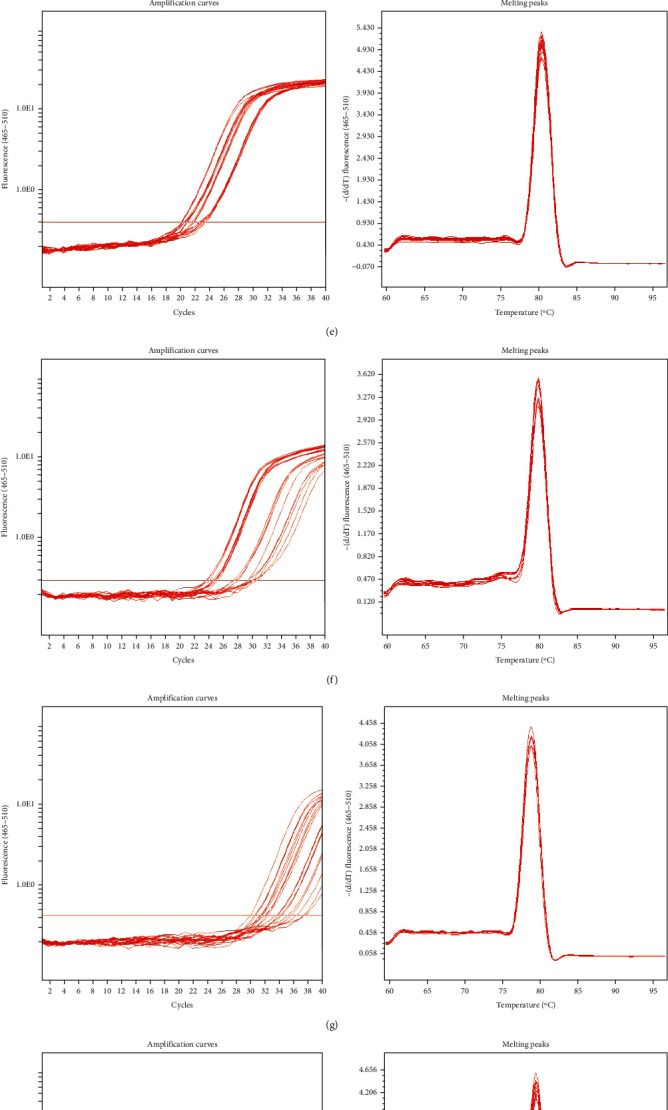
(a) Amplification curves and melting peaks of GAPDH. (b) Amplification curves and melting peaks of ENST00000436710. (c) Amplification curves and melting peaks of PVT1. (d) Amplification curves and melting peaks of TUG1. (e) Amplification curves and melting peaks of MALAT1. (f) Amplification curves and melting peaks of ENST00000505089. (g) Amplification curves and melting peaks of lnc-OR1M1-1 : 1. (h) Amplification curves and melting peaks of lnc-ST8SIA4-8 : 1. (i) Amplification curves and melting peaks of lnc-ZNF530-1 : 1. (j) Amplification curves and melting peaks of lnc-IGFBP3-1 : 1. (k) Amplification curves and melting peaks of ENST00000568998.

We also randomly selected 10 differentially expressed genes and further performed quantitative real-time polymerase chain reaction (qRT-PCR) to examine their expression levels (Table 2). The resulting melting curves all showed single peaks, with PCR amplification to show greater specificity (Figures 2(a)–2(k)).

### 3.2. GO Analysis and KEGG Analysis

**Figure 3:**
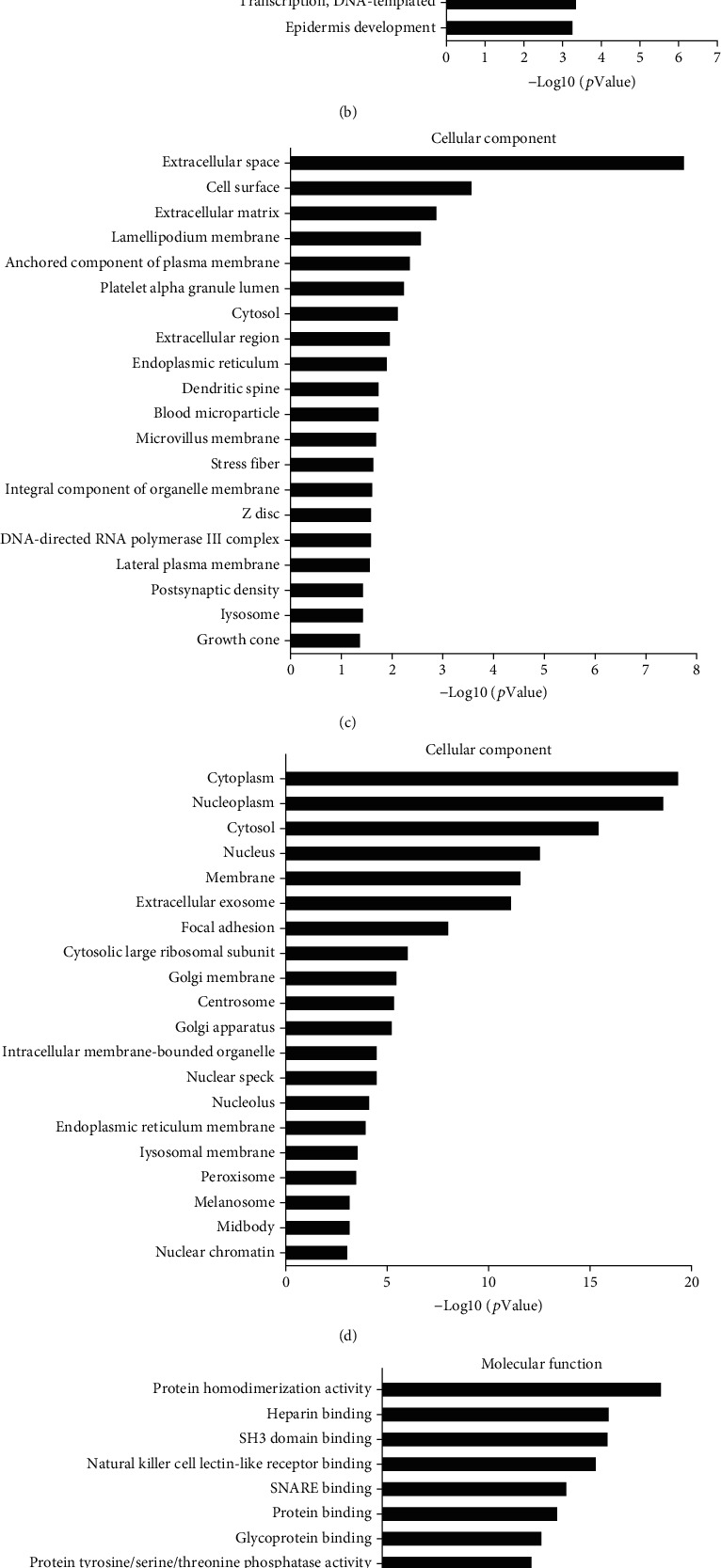
Gene Ontology (GO) enrichment analysis (top 20) in high-expression DNMT1 cell lines and normal esophageal epithelial cell lines: (a, b) biological process; (c, d) cellular component; (e, f) molecular function.

Gene Ontology (GO) analyses were conducted to explore the function of the 7421 differentially expressed mRNAs. The results showed that there are 1825 upregulated mRNAs expressed during various biological processes including those involved in blood coagulation, type I interferon signaling pathways, and response to viruses (Figure 3(a)). There were 3483 downregulated mRNAs in biological processes such as those involving viruses, SRP-dependent cotranslational proteins targeting membrane, and gene expression (Figure 3(b)). In terms of cellular components, there were 1937 upregulated mRNAs expressed including those associated with the extracellular space, the cell surface, and the extracellular membrane (Figure 3(c)). There were 3655 downregulated mRNAs expressed including those involved with cellular components such as the cytoplasm, nucleoplasm, and cytosol (Figure 3(d)). Evaluating molecular function, there were 1817 upregulated mRNAs detected that included those involved in protein homodimerization activity, heparin binding, and SH3 domain binding (Figure 3(e)). There were 3517 downregulated mRNAs expressed involving cellular components that included protein binding, poly (A) RNA binding, and ligase activity (Figure 3(f)).

**Figure 4:**
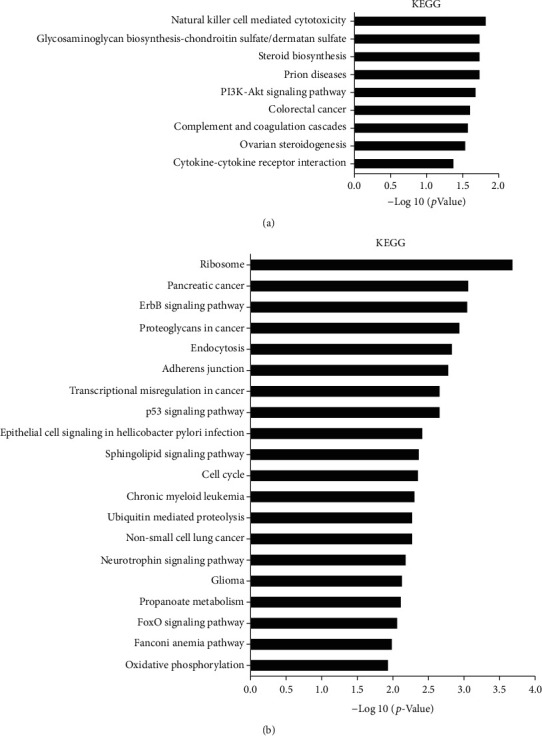
KEGG pathway analysis in high-expression DNMT1 cell lines and normal esophageal epithelial cell lines. (a) Nine pathways of upregulated mRNA enrichment; (b) the top 20 pathways enriched by downregulated mRNAs.

KEGG pathway analysis was conducted to examine the function of the 7421 differentially expressed mRNAs. The results showed that upregulated mRNAs were highly enriched in 9 gene pathways, including natural killer cell-mediated cytotoxicity, and glycosaminoglycan biosynthesis-chondroitin sulfate/dermatan sulfate and steroid biosynthesis (Figure 4(a)). Downregulated mRNAs were expressed in the 46 gene pathways including those involving ribosomes, pancreatic cancer, and the ErbB signaling pathway (Figure 4(b)).

### 3.3. lncRNA-mRNA Coexpression Networks

**Figure 5:**
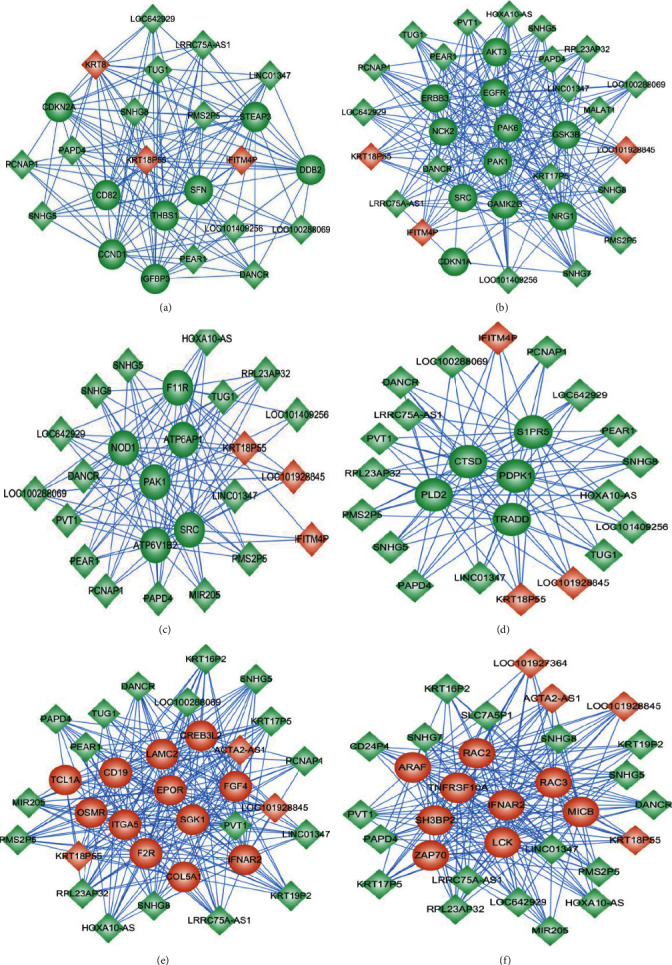
lncRNA-mRNA coexpression network in high-expression DNMT1 cell lines and normal esophageal epithelial cell lines. Round labels represent mRNA, square labels represent lncRNA, red indicates upregulation, and green indicates downregulation.

Based on the KEGG pathway results, we selected 6 pathways from the downregulated mRNA signaling and the upregulated mRNA signaling pathways to perform coexpression network analysis. In particular, we examined the p53 signaling and ErbB signaling pathways, respectively, as well as epithelial cell signaling in Helicobacter pylori infection, sphingolipid signaling pathway, PI3K-Akt signaling pathway, and natural killer cell-mediated cytotoxicity. Our results showed that 16 lncRNAs interacted with 8 mRNAs in the p53 signaling pathway (Figure 5(a)), 22 lncRNAs interacted with 11 mRNAs in the ErbB signaling pathway (Figure 5(b)), 19 lncRNAs interacted with 6 mRNAs in epithelial cell signaling in Helicobacter pylori infection (Figure 5(c)), 19 lncRNAs interacted with 5 mRNAs in the sphingolipid signaling pathway (Figure 5(d)), 21 lncRNAs interacted with 12 mRNAs in the PI3K-Akt signaling pathway (Figure 5(e)), and 22 lncRNAs interacted with 9 mRNAs in natural killer cell-mediated cytotoxicity (Figure 5(f)).

## 4. Discussion

DNMT1 can regulate the expression of genes in many different, complex ways. It mediates DNA methylation, modification of histones, and chromosome remodeling. As a result, a very complex epigenetic regulatory network is formed and regulates gene coexpression. In normal tissues, CpG islands in the gene promoter region are generally unmethylated. In tumor cells, the opposite is often true where CpG islands are hypermethylated which leads to silencing of their related genes [32, 33]. During replication, DNMT1 is localized in the replication complex and is associated with a methylated CpG island site in the parental chain which catalyzes the methylation gene then adds it to the corresponding CpG island site on the daughter strand. Validating DNMT1 location in differentiated cells allows comparison with the original methylation profile [34]. Studies have shown that hypermethylated genes are found in breast, colon, and stomach cancers [35–37]. Generally, DNMT1 expression increases before DNA methylation, which may cause abnormal DNA methylation.

lncRNAs have been the focus of a number of studies in recent years and have been found to be associated with the development of many types of tumors involving epigenetic, transcriptional, and posttranscriptional regulation during gene expression. While regulating DNA methylation, lncRNA mainly affects the expression of related genes by altering the methylation levels of CpG islands in the gene promoter region. Therefore, understanding lncRNA differential expression in the DNMT1 high-expression cell line and in normal esophageal epithelial cell line may be useful for understanding its function.

During coexpression analysis of the p53 signaling pathway, we found that lncRNA TUG1 is associated with the mRNA CDKN2A. TUG1 is widely expressed in various tumors and exhibits high expression levels in nervous system tumors, colorectal cancer, hematological system tumors, and bladder cancer. However, the expression level of TUG1 varies with different tissue types. It was found that compared with normal lung tissues/cells and paracancerous tissues, the expression of TUG1 in non-small-cell lung cancer tissues or cells was significantly reduced. This suggests that, on the one hand, 22 TUG1 may play a cancer-promoting role but and it can also play a role in inhibiting cancer [38]. In the present study, we found that TUG1 showed low expression levels in DNMT1 high-expression cells suggesting that TUG1 may be a tumor suppressor in this system. Khalil et al. [39] have demonstrated, using coimmunoprecipitation, that TUG1 recruits and binds to polycomb repressive complex 2 (PRC2) and PRC2 catalyzing the dimethylation of histone H3 at position 27. Trimethylation of lysine occurs at residue 27 of histone 3, H3 K27 me3 which, in turn, affects miRNAs, cyclin-dependent kinase inhibitors (e.g., p15, p16, p21, p27, and p57), and blood vessels which activate expression of related genes that participate in tumor development. CDKN2A is a cyclin-dependent kinase inhibitor that is located on human chromosome 9p21 and encodes two different proteins. One is a cell cycle-dependent kinase inhibitor p16^INK4*α*^ which is encoded by exons 1*α*, 2, and 3. The other is an alternate reading frame (ARF), encoded by exons 1*β*, 2, and 3 (in mice, called p19^ARF^), both of which are cellular regulators through cyclinD-CDK4-pRb-E2F and MDM2, respectively.

The p53 pathway is involved in cell cycle regulation [40]. Therefore, it may be inferred that TUG1 inhibits CKIs by recruiting PRC2 leading to excessive cyclinD-CDK4/6 kinase activation which may disrupt the cell cycle and promote cell proliferation. The loss of p16^INK4*α*^ leads to excessive activation of CDK4/6 kinase; however, modulation of the p16^INK4*α*^/pRB pathway will not inhibit the cancer. Much of this discussion, however, is speculative at this point and will require further verification both *in vivo* and *in vitro*.

lncRNA PVT1 was found to be related to CDKN2A mRNA in the ErbB signaling pathway. Further analysis of the function of lncRNA was provided by GO analysis. In addition, 19 lncRNAs interacted with 6 mRNAs in epithelial cell Helicobacter pylori infection signaling, 19 lncRNAs interacted with 5 mRNAs in the sphingolipid signaling pathway, 21 lncRNAs interacted with 12 mRNAs in the PI3K-Akt signaling pathway, and 22 lncRNAs interacted with 9 mRNAs in natural killer cell-mediated cytotoxicity. Therefore, our results suggest a key pathogenic role for lncRNAs.

Though this study had some limitations, valuable results were obtained from the bioinformatic and microarray analyses. However, further mechanistic studies will be needed to confirm the role of these differentially expressed genes and pathways. Nevertheless, this database serves as a valuable catalyst for further study.

## Figures and Tables

**Table 1:** Primer sequences.

Prime name	Forward	Reverse
lnc-OR1M1-1 : 1	CTGACATTTCAAGAGGTTGTGG	TGACTGATTCACTATTTGGTGC
lnc-IGFBP3-1 : 1	CTTCCTGGAGAGTCACTTCCTA	AGCCTTTCAAAGAGATACTCG
NR_003367(PVT1)	TTTCAGCACTCTGGACGG	AACACAGAGCACCAAGAC
ENST00000505089	CATCCTGATACCAAAGCCT	TTGATGTGTTGCTGGATTCG
ENST00000568998	CAAGGCTCCTCATAAGCA	GCACTTTGGGAGGTCAAT
lnc-ST8SIA4-8 : 1	ATGGTGACGTGATGTAATGC	TCTGAGGCGATAAATTGGACT
lnc-ZNF530-1 : 1	CGACCCAGGTATTATTGAGTG	TCAAACTCTTGGGCTCAAGG
NR_110492(TUG1)	TGGCTATTGGTATGGCTGG	TGACTGTAGTCCTCACGG
NR_002819(MALAT1)	CCTAAGGTCAAGAGAAGTGTC	GGTACTTCAAGCATTCCTTCG
ENST00000436710	CTTTGTCTTGGTGTCACCC	AGAACTTTCTCCACACGG
GAPDH	TGTTGCCATCAATGACCCCTT	CTCCACGACGTACTCAGCG

**Table 2:** Expression of lncRNAs in esophageal DNMT1 overexpressing cells and normal esophageal epithelial cells.

lncRNA	Experimental group	Control group	*T*	*P*
ENST00000436710	0.19 ± 0.18	1.48 ± 0.74	2.93	0.043
PVT1	1.73 ± 0.25	1.01 ± 0.21	3.80	0.019
TUG1	0.12 ± 0.12	1.05 ± 0.38	4.07	0.015
MALAT1	0.09 ± 0.003	1.01 ± 0.20	8.20	0.015
ENST00000505089	0.04 ± 0.04	1.08 ± 0.50	3.61	0.023
lnc-OR1M1-1 : 1	19.21 ± 8.97	1.02 ± 0.27	3.51	0.072
lnc-ST8SIA4-8 : 1	0.12 ± 0.04	1.02 ± 0.28	5.53	0.005
lnc-ZNF530-1 : 1	3.37 ± 0.43	1.05 ± 0.43	6.60	0.003
lnc-IGFBP3-1 : 1	3.47 ± 0.35	1.00 ± 0.09	11.90	0.000
ENST00000568998	0.19 ± 0.05	1.00 ± 0.09	13.47	0.000

## Data Availability

The microarray dataset has been uploaded to the GEO database. Data is available at NCBI GEO, accession number: GSE163735, https://www.ncbi.nlm.nih.gov/geo/.
